# Development of a Room Temperature SAW Methane Gas Sensor Incorporating a Supramolecular Cryptophane A Coating

**DOI:** 10.3390/s16010073

**Published:** 2016-01-07

**Authors:** Wen Wang, Haoliang Hu, Xinlu Liu, Shitang He, Yong Pan, Caihong Zhang, Chuan Dong

**Affiliations:** 1 State Key Laboratory of Acoustics, Institute of Acoustics, Chinese Academy of Sciences, No. 21, BeiSiHuan West Road, Beijing 100190, China; huhaoliang11@mails.ucas.ac.cn (H.H.); liuxinlu1987@foxmail.com (X.L.); heshitang@mail.ioa.ac.cn (S.H.); 2 State Key Laboratory of NBC Protection for Civilian, Yangfang, Changping District, Beijing 102205, China; panyjingyu2011@sina.com; 3 School of Chemistry and Chemical Engineering, Shanxi University, Taiyuan 030006, China; chzhang@sxu.edu.cn (C.Z.); dc@sxu.edu.cn (C.D.)

**Keywords:** surface acoustic wave, methane gas sensor, cryptophane A, room temperature, resonator-oscillator

## Abstract

A new room temperature supra-molecular cryptophane A (CrypA)-coated surface acoustic wave (SAW) sensor for sensing methane gas is presented. The sensor is composed of differential resonator-oscillators, a supra-molecular CrypA coated along the acoustic propagation path, and a frequency signal acquisition module (FSAM). A two-port SAW resonator configuration with low insertion loss, single resonation mode, and high quality factor was designed on a temperature-compensated ST-X quartz substrate, and as the feedback of the differntial oscillators. Prior to development, the coupling of modes (COM) simulation was conducted to predict the device performance. The supramolecular CrypA was synthesized from vanillyl alcohol using a double trimerisation method and deposited onto the SAW propagation path of the sensing resonators via different film deposition methods. Experiential results indicate the CrypA-coated sensor made using a dropping method exhibits higher sensor response compared to the unit prepared by the spinning approach because of the obviously larger surface roughness. Fast response and excellent repeatability were observed in gas sensing experiments, and the estimated detection limit and measured sensitivity are ~0.05% and ~204 Hz/%, respectively.

## 1. Introduction

Mining accidents with heavy casualties caused by methane gas explosions occur frequently, leading to huge economic losses. The most effective way to respond to such an issue is the early detection and monitoring of methane accumulation in mines or landfills. The current approaches for sensing methane gas include gas chromatography, electrochemical, optical, and semiconductor technologies. These techniques differ substantially in their approaches, and each have their own advantages and disadvantages [1,2,3,4]. Gas chromatography can perform an accurate quantitative analysis on methane gas, but, it is expensive and unsuitable for *in situ* monitoring which is essential in most cases [1]. Electrochemical methane gas sensors face greater challenges in real world applications because of the inertness of the methane molecule and their slow response [2]. The main challenge for optical methane sensors is that it is hard to find a suitable light source in the infrared range, and they also suffer from complicate sensor configurations and humidity interference [3,4,5]. As for the semiconductor methane gas sensors, their high operation temperature makes them unsuitable in mine environments due to the risk of explosions [6,7,8]. 

Therefore, the development of smart sensors with high sensitivity, fast response, excellent stability, and capable of room temperature operation is a necessary link for methane gas sensing and monitoring. The birth of the so-called surface acoustic wave (SAW) sensor technology opens up a new way for methane gas sensing. By means of sensitive materials with specific selectivity, SAW sensors have some excellent features such as high-sensitivity, ambient-temperature operation, simple packaging requirements, low cost, fast response, small size, and large dynamic range [9,10]. A typical SAW-based gas sensor configuration is composed of a differential oscillator array, and some sensitive materials deposited in sensing channels on the SAW propagation path of the SAW devices. The adsorption of the gas molecules to be analyzed by the sensitive interface modulates the SAW propagation properties. The corresponding change in velocity is read out by recording the frequency signal, which is directly proportional to the gas concentration. Obviously, the SAW device only plays a "quantification" role, while a qualitative assessment of the analyte is completed by suitable sensitive films. Unfortunately, it is not easy to find a good sensitive material candidate for sensing methane until the supramolecular species cryptophane A (CrypA) was synthesized and confirmed to show excellent selectivity to methane gas. As the smallest of the cryptophane family, Cryp A was utilized as the sensitive interface and exhibits an amazing affinity towards methane gas molecules by supramolecular interactions between the host and methane molecules [11,12], which arises from size complementarity and efficient van der Waals interactions. Recently, a quartz crystal microbalance (QCM)-based methane gas sensor configuration was presented employing CrypA as the sensitive interface [13,14], and superior selectivity, a fast response and a low detection limit of 0.05% were achieved at room temperature. However, the QCM-based methane gas sensor easily reaches saturation when the applied methane concentration is over 0.2%, that this makes it difficult to meet the actual requirements of underground methane gas alarms (the alarm point methane gas concentration is usually 1%).

**Figure 1:**
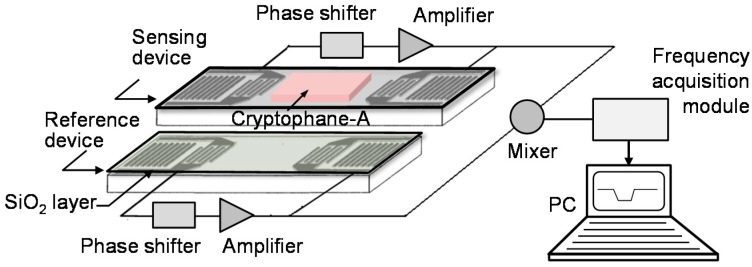
The schematic and working principle of the SAW-based methane gas sensor.

Hence, to address this issue, the main contribution in this work is to develop a new methane gas sensor incorporating SAW technology and CrypA films, which try to provide fast and accurate measurements in a larger dynamic range. The proposed sensor configuration consists of differential resonator-oscillators, and a sensitive coating on the sensing device surface, and a frequency signal acquisition module (FSAM), as shown in Figure 1. A two-port SAW resonator is designed on a temperature-compensated ST-X quartz substrate as the feedback of the differential oscillator. Lower insertion loss, high quality factor, and single resonation mode were achieved in the SAW devices developed by the photolithographic technique. For sensing methane gas, CrypA was synthesized from vanillyl alcohol and deposited onto the surface of the sensing SAW device. Different film deposition methods were applied for the CrypA coating to achieve higher sensor responses. The proposed SAW sensor was exposed to various concentrations of methane gas at room temperature, and the resulting performance, measured in terms of sensitivity, detection limit, and repeatability, was characterized experimentally.

## 2. Technique Realization

This section describes the realization of the physical structure of the CrypA-coated SAW methane sensor.

### 2.1. Two-Port SAW Resonator

A two-port SAW resonator configuration was reproducibly developed on a temperature-compensated ST-X quartz substrate as the oscillation feedback, as shown in Figure 1. 1600 Å Al-strip was deposited onto ST-X quartz wafer by using the photolithographic process. A thin SiO_2_ with 200 Å thickness is deposited on the device surface to protect the electrodes in the CrypA deposition by using plasma enhanced chemical vapor deposition (PECVD), and the SiO_2_ coating is amorphous and porous to increase the sensing contact area, which is benefitial for the interaction between the CrypA and methane gas. The design parameters of the SAW device are listed in Table 1. Prior to the SAW device development, the coupling of modes (COM) model was used for performance simulation.

**Table 1:** Design parameters for the SAW sensor chip.

Parameters	Values	Parameters	Values
Operation frequency (MHz)	300	Wavelength (λ: μm)	10.5
IDT length (λ)	41	Gap between the reflectors and IDT (λ)	0.75/0.5
Reflector length (λ)	300	Length of the coating area (λ)	150
aperture (λ)	200	Gap between the IDT and coating area (λ)	5

Using the typical admittance matrix solution for whole device, $\left[\text{Y}]=[\begin{array}{cc} y_{11} & y_{12}\\ y_{21} & y_{22} \end{array}\right]$, the frequency response *S*_21_ of the SAW device can be deduced by [15]:

(1) $$S_{21}=\frac{-2y_{12}\sqrt{G_{in}G_{out}}}{(G_{in}+y_{11})(G_{out}+y_{22})-y_{12}y_{21}}$$

**Figure 2:**
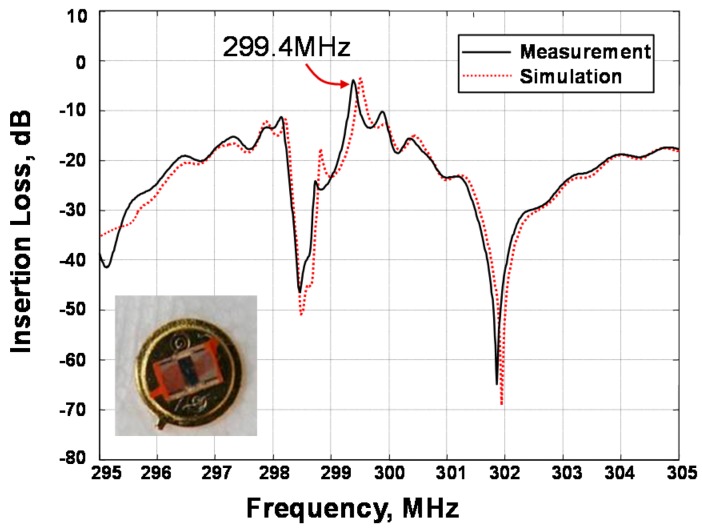
The measured and simulated frequency response of the SAW resonator.

Here, *G_in_* and *G_out_* are the input and output impedance, respectively. Using Equation (1), the SAW resonator with 1600 Å thick Al-strip was simulated utilizing the corresponding structure parameters listed in Table 1. The simulated frequency response of the SAW resonator is depicted in Figure 2. Low insertion loss of 4.5 dB, high quality factor of ~3500 and single resonation mode were achieved. Then, the fabricated SAW resonator (Figure 2) was characterized by using a network analyzer, and in comparison with the simulation. The measured result agrees well with the simulation. The resonant frequency of the developed SAW device is measured as 299.4 MHz.

### 2.2. Differential Resoantor-Oscillator

**Figure 3:**
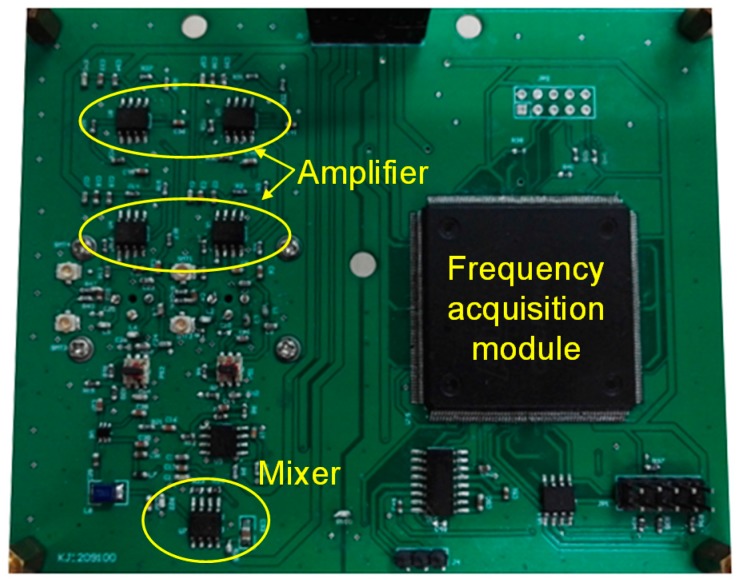
The oscillation PCB for methane gas sensor.

**Figure 4:**
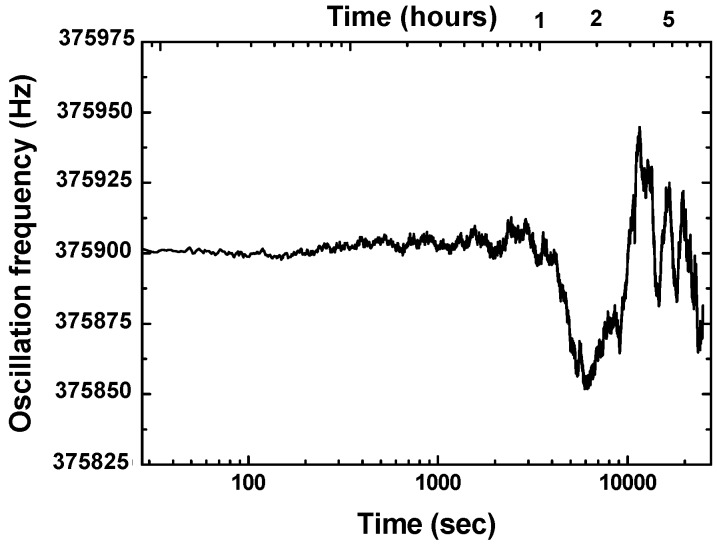
The measured frequency stabiltiy of the SAW oscillator.

The fabricated SAW sensor chip was loaded into a standard metal base (seen in the inset of Figure 2). As the feedback in the oscillation path, all transducers of the SAW resonators were connected by each oscillator circuit which was composed of an amplifier (BGA2817), phase shifter, mixer (UPC2758) and LPF and so on, as shown in Figure 3. The output of the each oscillator was mixed to obtain a different frequency in the MHz range and reduce the influence of the thermal expansion of the piezoelectric substrate. The mixed oscillation frequency signal was picked by a FPGA-based FSAM, and plotted in real-time by a self-made interface display program. Usually, the frequency stability of the oscillator affects significantly the detection limit of the gas sensor. Therefore, an experiment was conducted to measure the frequency stability of the developed differential resonator-oscillator at room temperature (20 °C) controlled by an incubator. To improve the frequency stability, the oscillation was conducted at the frequency point corresponding to the lowest insertion loss by a strategically phase modulation [15]. The measured short-term (in seconds) frequency stability of the oscillator without a sensor coating is ±1.5 Hz/s, and the medium term (in hours) frequency stability is better than ±25/h at the equilibrium status (Figure 4). One of the reasons for the frequency fluctuations is the environmental temperature which can only be stabilized to within ±0.5 °C.

### 2.3. Synthesis and Characterization of CrypA

**Scheme 1:**
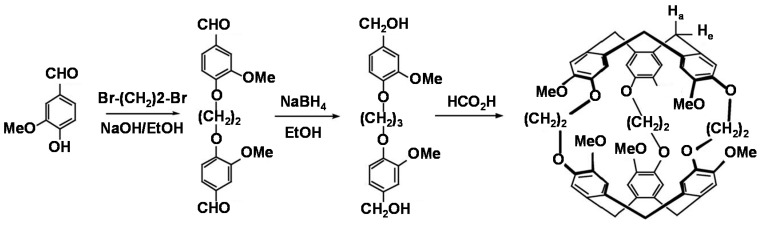
Synthesis of CrypA.

**Figure 5:**
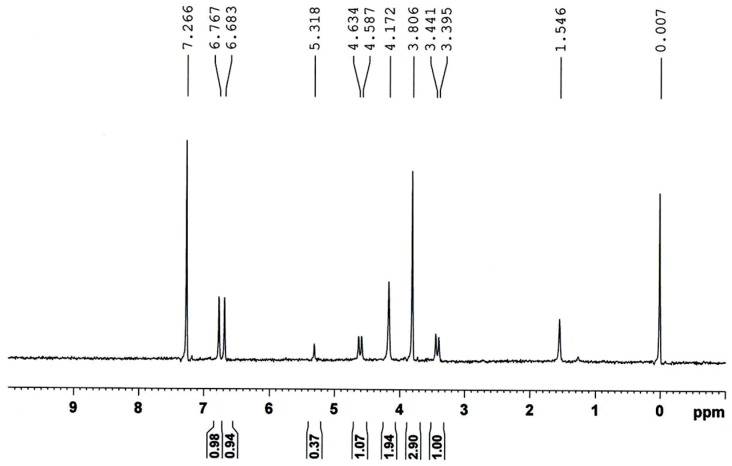
^1^H-NMR spectrum of the synthesized CrypA.

The synthesis of CrypA is depicted in Scheme 1, which was followed from the well-known two-step method [16]. Then, the synthesized CrypA was characterized by ^1^H-NMR (Figure 5), recorded in CDCl_3_ on a Bruker 400 MHz spectrometer and referenced to the residual solvent peak. The NMR results were: δ = 6.77 (s, 6H), 6.68 (s, 6H), 4.63 (d, *J =* 13.7 Hz, 6H), 4.17 (m, 12H), 3.81 (s, 18H), 3.44 (d, *J =* 13.8 Hz, 6H) ppm.

### 2.4. CrypA Deposition

Usually, the CrypA molecules are easily gathered and crystallized on the piezoelectric substrate surface, so the CrypA molecules are first dissolved in tetrahydrofuran (THF) prior to deposition on the sensing SAW device surface. The detailed composition of the CrypA-solution is that 3.0 mg CrypA, 0.3 mg polyvinyl chloride (PVC) and 0.6 mg dioctyl sebacate were dissolved in 2 mL THF. The polyvinyl chloride was used to improve the adhesion of CrypA. To determine the more effective way for CrypA formation, drop-coating and spin-coating were tested. Before the CrypA deposition, the SiO_2_ surface of the sensing SAW device was cleaned of any contaminants by a routine cleaning procedure involving rinsing in Piranha solution (V(H_2_SO_4_):V(H_2_O_2_) = 3:1), a DI water rinse and drying by N_2_. For drop-coating, 0.3 µL CrypA-solution was dropped on the cleaned SiO_2_ film surface between the IDTs of the sensing resonator, and then cured at 80 °C for 40 min in an oven. For spin-coating, 100 µL solutions were spin at 2000 rpm for 30 s on a ST-quartz wafer with SAW resonator patterns. The wafer was also cured at 80 °C for 40 min and then diced into individual SAW resonators.

**Figure 6:**
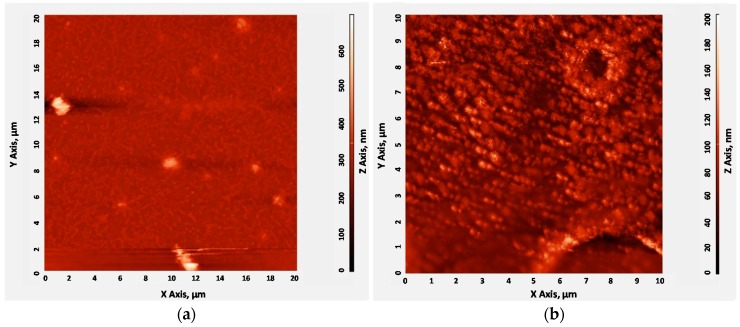
AFM characterization of CrypA coated by (**a**) Spin-coating; (**b**) Drop-coating.

Then, the surface topography of CrypA-coated sensor chips utilizing different CrypA coating methods was characterized by the atomic force microscope (AFM), as shown in Figure 6. As a rough estimate, the thicknesses of the CrypA coating deposited by spin-coating and drop-coating are ~600 nm and ~200 nm, respectively. It is also obviously that the CrypA film formed by drop-coating has an obviously larger rougher surface with many fluctuations and bubbles, whereas the film surface coated with spin-coating is much smoother.

## 3. Sensor Experiments

### 3.1. Gas Sensor Experimental Setup

**Figure 7:**
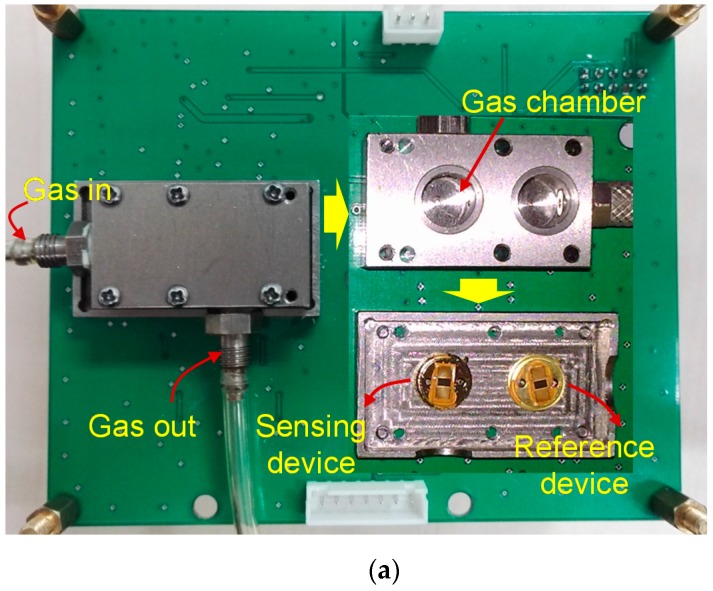

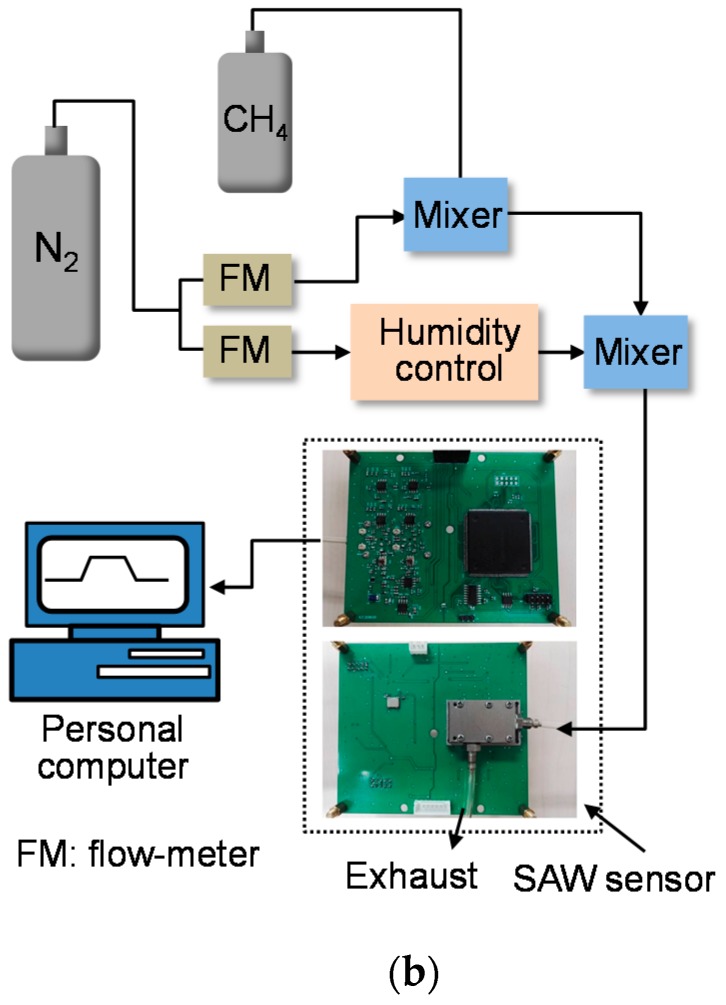
The gas chamber (**a**) and experimental set up (**b**) of the sensor system.

The developed CrypA-coated sensing chip and reference chip were placed in a surface nickel-plated Aluminum gas chamber with volume of 500 mL(Figure 7a), and connected to corresponding oscillation circuit, respectively. The experimental set up in Figure 7b was utilized to characterize the sensor responses towards methane gas at room temperature. The SAW sensor was exposed to N_2_ and CH_4_ gas alternately via the gas path, as shown in Figure 7b. To study the humidity effects, air was used as the diluents, and the relative humidity (RH) was controlled by a streaming standard humidity generator (RST-GX-2, Beijing Naisisa New Technology Development Corp, Beijing, China.). The sensor signal were collected at 60 points per minute by the FSAM, and plotted by the personal computer in real time.

### 3.2. Sensor Performance Evaluation

**Figure 8:**
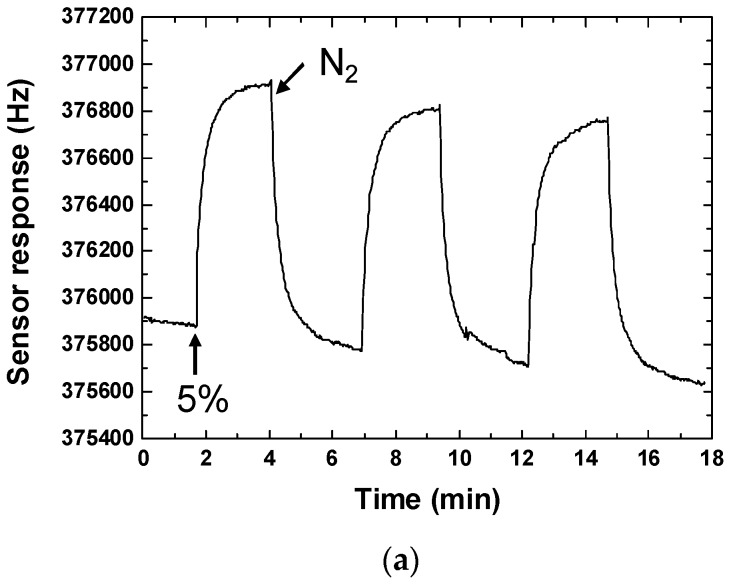

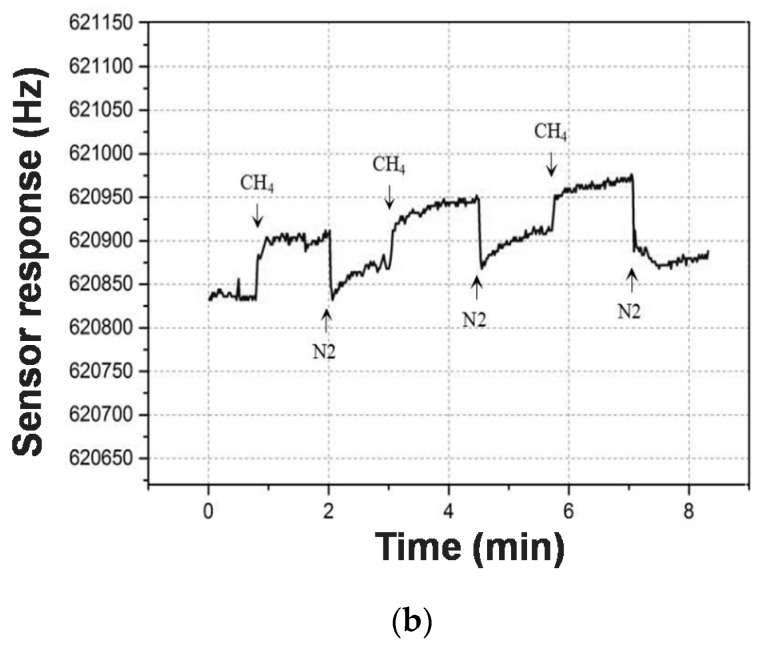
Sensor response and repeatability testing towards 5% CH_4_ from the SAW sensor with different CrypA coating method. (**a**) Dropping-method; (**b**) Spinning-coating.

First, the repeatability of the developed CrypA-coated SAW sensor was evaluated. Figure 8 showed a response profile obtained from three consecutive 18 min on-off exposures to 5% of CH_4_ in pure N_2_ at 20 °C using the developed sensors with CrypA deposited by different coating approaches. ~1 kHz of frequency response was achieved from the sensor with CrypA coated by drop-coating method (Figure 8a). It can also be noted that three gas exposures are in good reproducible run. The gathered frequency signal showed a rapid rise upon exposure to CH_4_ and reaches approximately the equilibrium (saturation) value in 12 s. When the gas was removed by N_2_ injection, the sensor response returned to its initial baseline within 20 s. It means the 90% response time of ~12 s and recovery time of ~20 s with good repeatability were obtained at room temperature. These promising results indicated that this sensor exhibits fast response and excellent repeatability in response to CH_4_. The sensor response towards 5% CH_4_ was also conducted from the sensor with CrypA coated by spinning-coating, as shown in Figure 8b, only ~100 Hz of frequency response was observed, much smaller than that of drop-coating CrypA sensor. Considering the CrypA film topography characteristics mentioned above, the experimental results indicate that the surface roughness of CrypA coating influences greatly on the sensor response. Sensitive film with larger rougher surface provides larger surface-to-volume ratio, thus more CrypA molecules are able to contact and absorb methane gas, resulting in higher sensor response.

**Figure 9:**
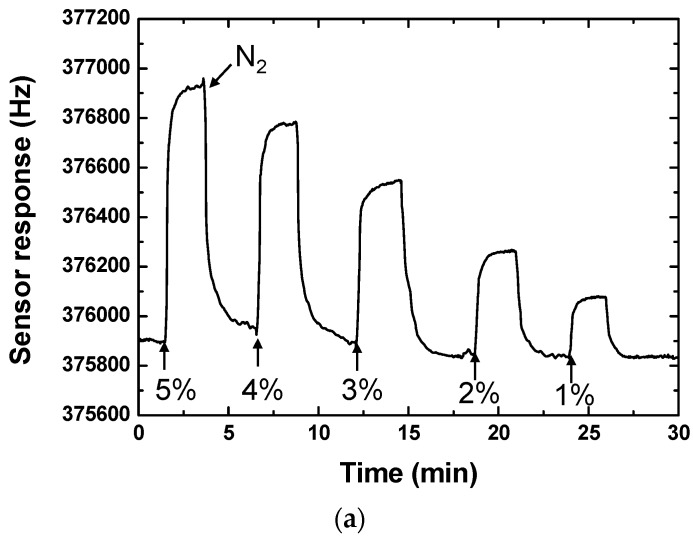

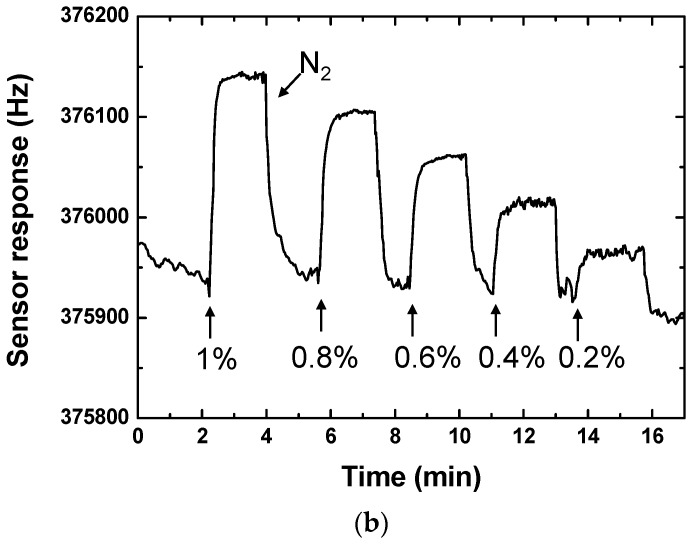
Responses of CrypA based SAW sensor to (**a**) High CH_4_ concentrations and (**b**) Low CH_4_ concentration at room temperature.

**Figure 10:**
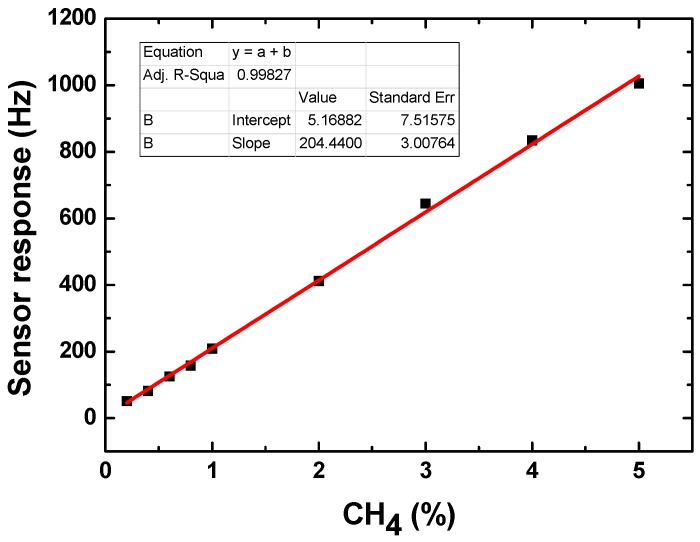
Frequency shifts of CrypA-coated SAW sensor *vs.* different concentrations of CH_4_.

Then, we exposed the CrypA-coated sensors by using the drop-coating to CH_4_ with different concentrations to characterize their sensitivity. Figure 9 shows the real-time response of the CrypA sensor to low (Figure 9a) and high (Figure 9b) CH_4_ concentrations at room temperature (20 °C). And the maximum frequency response was plotted *vs.* the corresponding CH_4_ concentrations as shown in Figure 10. It is obviously that as the gas concentration increased, the sensor frequency signal also increased with approximately linearity. The sensitivity in CH_4_ concentration range of 0.2%~5% was evaluated as ~204 Hz/% with well linearity of 0.99827. Also, from Figure 9b, the sensor response of ~50 Hz occurs at CH_4_ concentration of 0.2%. It means lower detection limit will be expected because the present oscillator exhibits excellent short-term frequency stability of ±1.5 Hz/s. Hence, based on the International Union of Pure and Applied Chemistry (IUPAC) (Zurich, Switzerland), the detection limit of the developed sensor can be estimated to less than 0.05%, the same rank to the reported CrypA-coated QCM sensor but the dynamic range is larger more [9]. The measure results indicate that the presented CrypA-coated SAW sensor was very promising for CH_4_ detection and monitor in homes, industries and mines.

### 3.3. Humidity Effect Evaluation

**Figure 11:**
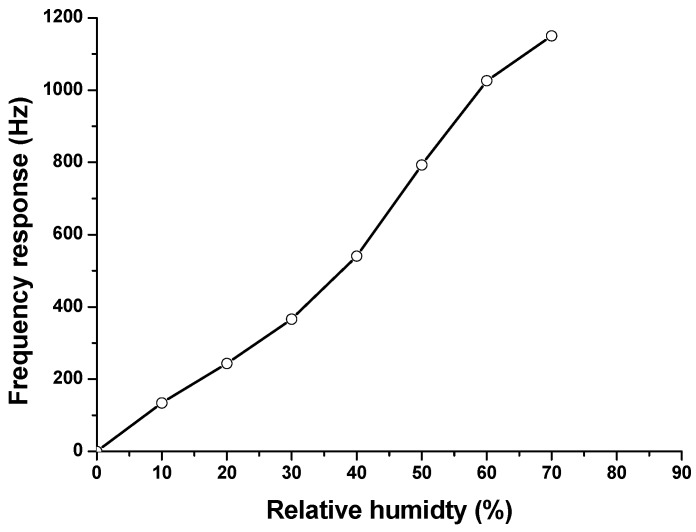
Frequency response of the SAW sensor to relative humidity.

Usually, the gas sensors are usually deployed in the ambient dynamic environment that may affect significantly the sensor performance [17]. Therefore, it is necessary to study the humidity effect on these sensors. Figure 11 illustrated the crossed humidity sensitivity of the developed SAW sensor, it clearly indicates that the sensor signal increased as the RH increased. The response to a constant methane concentration of 5% but different humidity levels of 0%, 20%, and 30% was 1015 Hz, 1258 Hz, and 1381 Hz, respectively. Obviously, the humidity affected the sensor performance significantly. The reason for the humidity effect is the adsorption of water molecules in CrypA while methane sensing. As described in Reference [10], the humidity effect on sensor response can be alleviated in different ways like calibration by creating a special database relating to different humidity level, or utilizing a polymer coating on the reference device to estimate the humidity effect by difference method.

## 4. Conclusions

A room temperature SAW sensor incorporating a CrypA coating was developed for sensing methane gas. The two-port SAW resonsators with low insertion loss, high quality factor, and single resonation mode were fabricated on a temperature-compensated ST-X quartz substrate as the feedback element in oscillation path. The synthesized Supramolecular CrypA was deposited onto the sensing SAW device as the sensitive material for sensing methane gas. Different CrypA film deposition approaches were conducted to achieve higher sensor response. The sensor responses to methane were evaluated experimentally. Fast response and excellent repeatability were observed, and the estimated detection limit and sensitivity are ~0.05% and ~204 Hz/%, respectively.
